# 22q11.2 deletion syndrome and psychosis – regarding a clinical case

**DOI:** 10.1192/j.eurpsy.2022.1980

**Published:** 2022-09-01

**Authors:** F. Santos Martins, C. Guerra

**Affiliations:** 1 Centro Hospitalar Universitário São João, Psychiatry And Mental Health Department, Porto, Portugal; 2 Faculty of Medicine, University of Porto, Clinical Neurosciences And Mental Health Department, Porto, Portugal

**Keywords:** high-risk psychosis, Psychosis, 22q11.2 deletion syndrome, DiGeorge syndrome

## Abstract

**Introduction:**

22q11.2 deletion syndrome is the most common microdeletion syndrome. Its clinical presentation varies and it may present several medical complications, namely heart defects, cleft palate, autoimmune diseases, delayed development, and psychiatric disorders. In these patients, psychiatric disorders are frequent and may include attention-deficit hyperactivity disorder, anxiety disorders, autism spectrum disorder, and schizophrenia spectrum disorders.

**Objectives:**

We aim to characterize psychosis in patients diagnosed with 2q11.2 deletion syndrome, which is one of the most frequent psychiatric presentations.

**Methods:**

To introduce the topic of 22q11.2 psychiatric symptoms, we will start by presenting a clinical case. Then, a review of the related literature using the *Pubmed* database using the following expression “22q11.2 deletion syndrome”; “DiGeorge syndrome”; “velocardiofacial syndrome”; “psychosis”; “psychiatric disorders”.

**Results:**

Patients diagnosed with 22q11.2 deletion syndrome are considered high-risk for psychosis. In this clinical case, we present a 19-year old man diagnosed with 22q11.2 deletion syndrome who was admitted to a psychiatric ward for psychosis. The knowledge of the increased risk for psychosis in these patients should be taken into account in the face of behavioral changes de novo to assure a timely therapeutic approach. Currently, the treatment does not differ from other patients, but this is mainly due to the lack of knowledge on the best therapeutic approach in this specific diagnosis.

**Conclusions:**

Genetic syndromes are often associated with psychiatric disorders. Patients diagnosed with 22q11.2 deletion syndrome are at high risk for psychosis and should deserve a multidisciplinary approach so that their diagnosis and treatment are established as early as possible.

**Disclosure:**

No significant relationships.

